# Soluble suppression of tumorigenicity 2 as outcome predictor after cardiopulmonary resuscitation: an observational prospective study

**DOI:** 10.1038/s41598-021-01389-x

**Published:** 2021-11-05

**Authors:** Richard Rezar, Vera Paar, Clemens Seelmaier, Ingrid Pretsch, Philipp Schwaiger, Kristen Kopp, Reinhard Kaufmann, Thomas K. Felder, Erika Prinz, Geza Gemes, Rudin Pistulli, Uta C. Hoppe, Bernhard Wernly, Michael Lichtenauer

**Affiliations:** 1 Clinic of Internal Medicine II, Department of Cardiology, Paracelsus Medical University of Salzburg, Salzburg, Austria; 2 Department of Radiology, Paracelsus Medical University of Salzburg, Salzburg, Austria; 3 Department of Laboratory Medicine, Paracelsus Medical University of Salzburg, Salzburg, Austria; 4 Department of Anaesthesiology and Intensive Care Medicine, Krankenhaus Der Barmherzigen Brüder Graz, Graz, Austria; 5 Department of Cardiology I-Coronary and Peripheral Vascular Disease, Heart Failure, University Hospital Münster, Münster, Germany; 6 Department of Anaesthesiology, Perioperative Medicine and Intensive Care Medicine, Paracelsus Medical University of Salzburg, Salzburg, Austria; 7 Center for Public Health and Healthcare Research, Paracelsus Medical University of Salzburg, Salzburg, Austria

**Keywords:** Biomarkers, Cardiovascular diseases

## Abstract

Prognostication after cardiopulmonary resuscitation (CPR) is complex. Novel biomarkers like soluble suppression of tumorigenicity 2 (sST2) may provide an objective approach. A total of 106 post-CPR patients were included in this single-center observational prospective study. Serum sST2 levels were obtained 24 h after admission. Individuals were assigned to two groups: patients below and above the overall cohort’s median sST2 concentration. Primary outcome was a combined endpoint at 6 months (death or Cerebral Performance Category > 2); secondary endpoint 30-day mortality. A uni- and multivariate logistic regression analysis were conducted. Elevated sST2-levels were associated with an increased risk for the primary outcome (OR 1.011, 95% CI 1.004–1.019, p = 0.004), yet no patients with poor neurological outcome were observed at 6 months. The optimal empirical cut-off for sST2 was 46.15 ng/ml (sensitivity 81%, specificity 53%, AUC 0.69). Levels above the median (> 53.42 ng/ml) were associated with higher odds for both endpoints (death or CPC > 2 after 6 months: 21% vs. 49%, OR 3.59, 95% CI 1.53–8.45, p = 0.003; death after 30 days: 17% vs. 43.3%, OR 3.75, 95% CI 1.52–9.21, p = 0.003). A positive correlation of serum sST2 after CPR with mortality at 30 days and 6 months after cardiac arrest could be demonstrated.

## Introduction

Prognostication after cardiac arrest (CA) remains a multi-faceted challenge in modern intensive care medicine. Various clinical parameters, neurophysiological tests, as well as imaging and laboratory findings can be included in the decision-making process^1^. Novel cardiovascular biomarkers are the subject of current research and could provide an objective approach in prognostication after successful cardiopulmonary resuscitation (CPR). Current guidelines emphasize uncertainty regarding ultimate cut-off values for conventional neuroprognostication markers such as neuron-specific enolase (NSE) and S-100B protein^2^. Furthermore, the use of glial fibrillary acidic protein, serum tau protein or neurofilament light chain remains subject of controversial debate^2^.

Suppression of tumorigenicity 2 (ST2) is assumed to play a role in inflammatory T-cell mediated processes^3^. In recent years, the importance of the ST2-/IL-33 axis has been repeatedly investigated for patients with different cardiovascular or inflammatory diseases^4–6^. Various pro-inflammatory but also protective effects (especially regarding cardiovascular remodeling) have been attributed to these signaling pathways^4,7^. Significantly divergent biomarker concentrations have been shown to be prognostically relevant for various disease entities^8–11^.

Data on sST2-levels after resuscitation are scarce, hence further investigation is reasonable and necessary. Ristagno and colleagues demonstrated that sST2 and circulating, complement-activating pattern-recognition receptor Pentraxin 3 (PTX3) were independently associated with mortality and multiorgan failure immediately after ICU admission in patients in the FINNRESUSCI collective. In addition, sST2-levels 48 h after admission were found to be independently associated with ICU mortality^12^. In the past, we have investigated the sST2/IL-33 axis in different cardiovascular diseases and gained good experience with sST2 as a powerful indicator of generalized inflammation as well as a prognostic parameter. In our single-center observational study, we prospectively investigated the prognostic relevance of the sST2/IL-33-axis, measured 24 h after cardiopulmonary resuscitation to create thesis-generating data for large future studies.

## Patients and methods

### Study subjects

**Figure 1:**
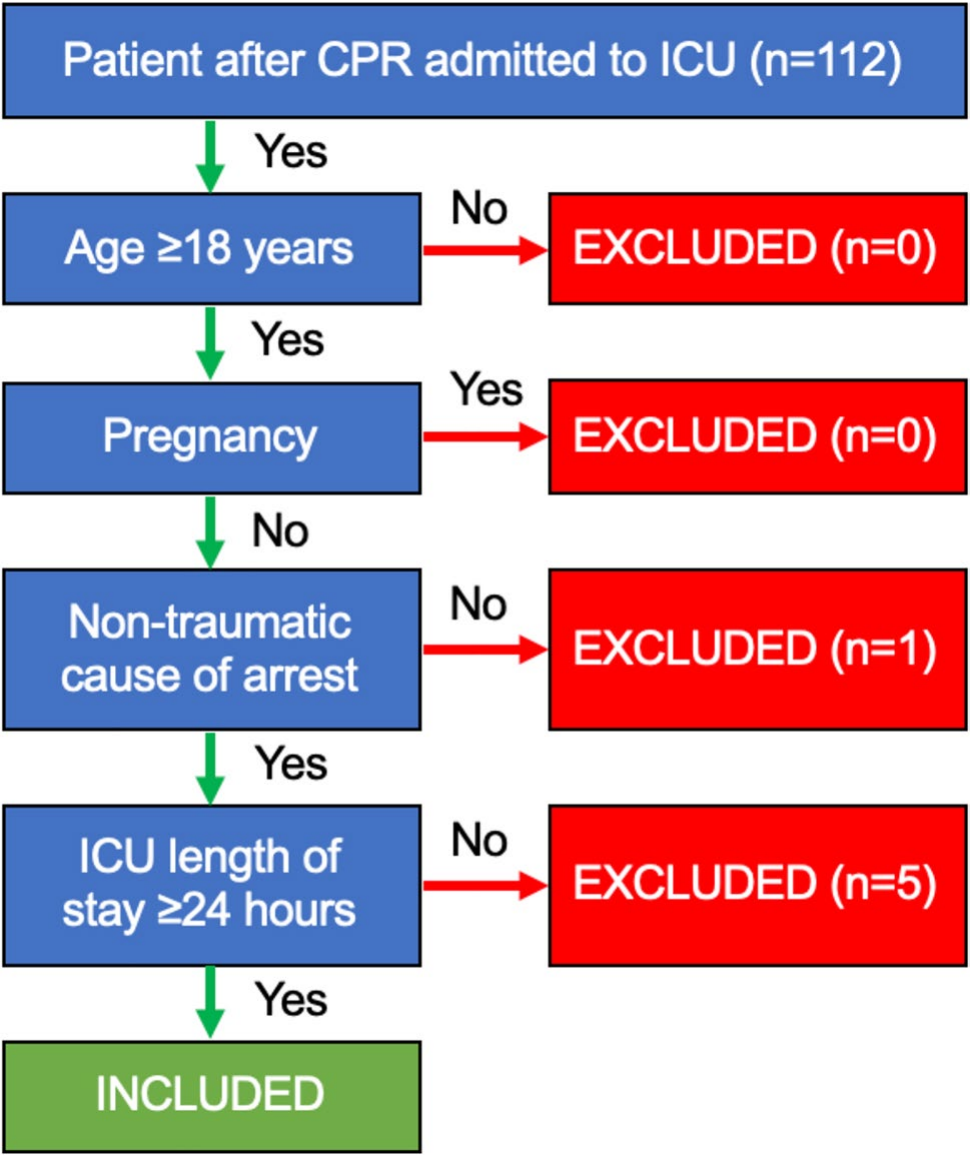
Patient enrollment process. *CPR* cardiopulmonary resuscitation, *ICU* intensive care unit.

This observational, prospective, single-center study was conducted at the medical ICU at the Paracelsus Medical University Salzburg between December 2018 and March 2020. All patients with a minimum age of 18 years admitted after cardiopulmonary resuscitation (both out-of-hospital and in-hospital CA) with a minimum stay of 24 h were included, after exclusion of traumatic cardiac arrest. Pregnant patients were excluded from the study. The process of patient enrollment is shown in Fig. 1. The study was conducted according to the principles of the Declaration of Helsinki and Good Clinical Practice and approved by the local ethics committee (Ethikkommission Land Salzburg 415-E/2408/8-2018). Written informed consent was obtained; in deceased patients or patients with poor neurological outcome, informed consent was obtained from next of kin when applicable. All patients received optimal standard care regardless of study participation. Laboratory tests and imaging studies were performed independently of study inclusion according to local practice guidelines. Left ventricular ejection fraction was determined by echocardiography by the attending physician immediately after admission to the ICU. It was determined to be “normal” (in males: 52–72%, in females 54–74%), “mildly abnormal” (in males: 41–51%, in females 41–53%), “moderately abnormal” (in both sexes 30–40%) and “severely abnormal” (in both sexes < 30%) according to the 2015 ASE Recommendations for Cardiac Chamber Quantification by Echocardiography in Adults^13^. Medical personnel were blinded to the determined sST2-values. Follow-up examinations included neurologic assessment via Cerebral Performance Category (CPC) and were performed by telephone at 30 days and 6 months.

### Laboratory analysis

Blood samples for the study were collected 24 h after ICU admission along with routine probes. Serum tubes were used for analysis of novel cardiovascular biomarkers and centrifuged thirty minutes after blood collection. Thereafter, the supernatant was frozen at minus 20 °C. Within 1 month after collection, laboratory samples were stored at minus 80 °C to avoid loss of quality according to practice guidelines of our research laboratory. Standard laboratory tests were performed according to guidelines of our hospital’s main laboratory. The serum concentrations of sST2 and IL-33 were analyzed using commercially available enzyme-linked immunosorbent assay (ELISA)-kits (Human ST2/IL-33R DuoSet ELISA DY523B-05 and Human IL-33 DuoSet ELISA DY3625B, R&D Systems, USA). Reagent preparation and measurements were performed according to the manufacturer’s instructions. In brief, the plates were coated with the appropriate capture antibody. Then the patient blood samples and the standard protein were added to the wells of the ELISA plates (Nunc MaxiSorp, flat-bottom 96 well plates, VWR International GmbH, Austria). An initial incubation time was set at two hours. Plates were then washed with phosphate-buffered saline (PBS) solution with 0.05% Tween 20 (Tween 20, Carl Roth, Germany) and then a biotin-labelled antibody was added. The plates were then incubated again for two hours, then washed again and a Streptavidin-horseradish-peroxidase solution was added. Tetramethylbenzidine (TMB; Sigma Aldrich, USA) was applied as a substrate to induce a color reaction. After a maximum of 20 min, the color reaction was stopped by adding 2N sulfuric acid (H_2_SO_4_). The optical density (OD) of the samples and standards was determined using a microplate-reader at a wavelength of 450 nm (iMark Microplate Absorbance Reader, Bio-Rad Laboratories, Austria).

### Statistical analysis

Continuous data points were expressed as median ± interquartile range. Differences between independent groups were calculated accordingly using Mann Whitney U-test. Categorical data were expressed as numbers (percentage). A Chi-square test was used to calculate univariate differences between groups. First, we divided the cohort into two sub-groups: patients below and patients above the median sST2 concentration of this cohort. The primary outcome was a composite endpoint after 6 months (death or CPC > 2); the secondary endpoint death after 30 days. Also, we stratified the cohort by sST2-level quartiles to investigate a graded association of sST2 level and prognosis. In addition, the associations of sST2 as a continuous variable and as a binary variable (above and below the median) were evaluated using logistic regression. Also, an optimal cut-off was calculated by means of the Youden index. Receiver operating curve (ROC) analysis was performed, and the area under the curve (AUC) calculated. We created a univariable model as well as a multivariable model in which confounding factors (age, sex and sequential organ failure assessment (SOFA) score) were included and calculated odds ratios (OR) as well as adjusted odds ratios (aOR) with their respective 95% confidence intervals (95% CI). The selection of confounding factors was based on our clinical experience as well as previous research. To investigate the association of being in the high-ST2 group and the secondary outcome, a univariate Cox proportional hazard model was used and hazard ratios (HR) were calculated. The survival curve was plotted using the Kaplan Meier curve. All tests were two-sided and a p-value < 0.05 was considered significant. We used Stata 16.1 (Stata Statistical Software: Release 16. StataCorp LLC, College Station, Texas, United States of America) for all statistical calculations.

### Ethics approval and consent to participate

The study was conducted according to the principles of the Declaration of Helsinki and Good Clinical Practice and approved by the local ethics committee (415-E/2408/8-2018).

## Results

A total of 106 patients were included in the final analysis. The cohort was divided into sST2 levels below or above the median for further analysis. The median sST2 level 24 h after ICU admission was 53.42 ng/ml (IQR 34.68–93.57 ng/ml) for the entire cohort. There were no significant differences in age, sex, body mass index, smoking history or pre-existing chronic disease except for coronary artery disease (CAD). Patients with sST2-levels below the median were more likely to have a history of CAD (24.5% vs. 9.4%, p = 0.038). Furthermore, no significant correlation could be shown for ejection fraction determined by transthoracic echocardiography. In Table 1 a detailed overview of general patient characteristics is provided. Supplementary Table 1 (Supplement 1) shows the results within quartiles of sST2 levels.

**Table 1:** General patient characteristics.

Characteristic	sST2 ≤ 53.42 ng/ml (n = 53)	sST2 > 53.42 ng/ml (n = 53)	p-value
Age (yrs.)—median (IQR)	65 (57–74)	64 (53–72)	0.444
Male sex—no. (%)	38 (71.7)	38 (71.7)	1.000
BMI (kg/m_2_)—median (IQR)	25.6 (24.6–28.2)	26.2 (24.5–29.1)	0.675
Arterial hypertension—no. (%)	37 (69.8)	34 (64.2)	0.708
Hyperlipidemia—no. (%)	33 (62.3)	24 (45.3)	0.076
Diabetes mellitus—no. (%)	7 (13.2)	12 (22.6)	0.170
Smoking history—no. (%)	24 (45.3)	26 (49.0)	0.637
COPD—no. (%)	7 (13.2)	6 (11.4)	0.767
OSA—no. (%)	5 (9.4)	2 (3.8)	0.241
CKD—no. (%)	11 (20.8)	9 (17.0)	0.653
History of CAD—no. (%)	13 (24.5)	5 (9.4)	0.038
History of cardiac surgery—no. (%)	5 (9.4)	3 (5.7)	0.594
Ejection fraction after admission—no. (%)	–	–	0.429
Normal^(1)^	9 (17.0)	8 (15.1)	–
Mildly abnormal^(2)^	9 (17.0)	16 (30.2)	–
Moderately abnormal^(3)^	20 (37.7)	15 (28.3)	–
Severely abnormal^(4)^	15 (28.3)	14 (26.4)	–

*BMI* body mass index, *CAD* coronary artery disease, *CKD* chronic kidney disease, *COPD* chronic obstructive pulmonary disease, *IQR* interquartile range, *no.* number, *OSA* obstructive sleep apnea, *yrs.* years.

Ranges of percentage for left ventricular ejection fraction: (1) “normal”: males 52–72%, females 54–74%; (2) “mildly abnormal”: males 41–51%, females 41–53%; (3) “moderately abnormal”: both sexes 30–40%; (4) “severely abnormal”: both sexes < 30%.

Regarding CPR-specific findings there was no difference between the two groups in the proportion of out-of-hospital cardiac arrests (OHCA) and in-hospital cardiac arrests (IHCA) (84.9% each, p = 1.000). No data were collected on first medical contact (paramedic vs. physician-based emergency medical service; EMS), yet all patients with OHCA were eventually admitted to our institution by physician-based EMS vehicles. Emergency response teams for IHCA were also led by physicians. Significantly more patients in the lower sST2 group received bystander CPR (49.0% vs. 28.3%, p = 0.040) whereas more patients in the higher sST2 group received CPR during transport (13.2% vs. 3.8%, p = 0.040). No relevant difference was found for initial rhythm, direct admission to the ICU or percutaneous coronary intervention (PCI). More patients with sST2-levels above the median received prehospital systemic lysis, which also did not reach statistical significance (11.4% vs. 5.7%, p = 0.296). Regarding post resuscitation care, patients in the group with higher sST2 levels more often received mechanical ventilation (100% vs. 88.7%, p = 0.012), targeted temperature management (83% vs. 54.7%, p = 0.002), continuous veno-venous hemodiafiltration (18.9% vs. 0%, p = 0.001) and antimicrobial chemotherapy (100% vs. 84.9%, p = 0.001), while no statistically significant difference but a trend towards more red cell blood transfusions was shown in the higher sST2-group (15.1% vs. 9.4%, p = 0.374). Table 2 provides a detailed overview of the characteristics associated with CPR- and post-resuscitation care. Supplementary Table 2 (Supplement 1) shows the results within quartiles of sST2 levels.

**Table 2:** Patient characteristics regarding CPR and post-resuscitation care.

Characteristic	sST2 ≤ 53.42 ng/ml (n = 53)	sST2 > 53.42 ng/ml (n = 53)	p-value
OHCA—no. (%)	45 (84.9)	45 (84.9)	1.000
**Bystander CPR—no. (%)**	–	–	0.040
Yes	26 (49.0)	15 (28.3)	–
No	4 (7.5)	10 (18.9)	–
In-hospital	7 (13.2)	7 (13.2)	–
During transport	2 (3.8)	7 (13.2)	–
Unknown	14 (26.4)	14 (26.4)	–
**Initial rhythm**	–	–	0.347
Ventricular fibrillation	39 (73.6)	37 (69.8)	–
Asystole	7 (13.2)	8 (15.1)	–
PEA	3 (5.7)	8 (15.1)	–
Unknown/other	4 (7.5)	0 (0.0)	–
**Admission directly to ICU—no. (%)**	–	–	0.807
Yes	12 (22.6)	13 (24.5)	–
Via ER	34 (64.2)	31(58.5)	–
Via Cath-lab	7 (13.2)	9 (17.0)	–
Systemic lysis—no. (%)	3 (5.7)	6 (11.4)	0.296
Coronary angiography—no. (%)	43 (81.1)	38 (71.7)	0.253
PCI—no. (%)	29 (54.7)	30 (56.6)	0.845
Mechanical ventilation—no. (%)	47 (88.7)	53 (100.0)	0.012
TTM—no. (%)	29 (54.7)	44 (83.0)	0.002
CVVHDF—no. (%)	0 (0.0)	10 (18.9)	0.001
Antibiotics—no. (%)	45 (84.9)	53 (100.0)	0.001
Blood transfusions—no. (%)	5 (9.4)	8 (15.1)	0.374
SOFA-Score—median (IQR)	10 (8–11)	12 (10–12)	< 0.001

*CPR* cardiopulmonary resuscitation, *CVVHDF* continuous veno-venous hemodiafiltration, *ER* emergency room, *ICU* intensive care unit, *IQR* interquartile range, *OHCA* out of hospital cardiac arrest, *PCI* percutaneous coronary intervention, *PEA* pulseless electrical activity, *SOFA* severity of organ failure assessment, *TTM* targeted temperature management.

Standard laboratory values showed a significant difference in initial arterial pH- and lactate-values after ICU admission (high-sST2: median pH 7.151, median lactate 5.74 mmol/l vs. low-sST2: median pH 7.257, median lactate 3.58 mmol/l; p = 0.003 each). In addition, patients with higher initial sST2-levels had higher initial leucocyte counts (15.6 G/l vs. 12.6 G/l, p = 0.002). No relevant differences were observed in initial hemoglobin, platelet-count, C-reactive protein (CRP), serum creatinine, low-density lipoprotein (LDL) or HbA1c. A detailed list of all obtained laboratory values is provided in Table 3. Supplementary Table 3 (Supplement 1) shows the results within quartiles of sST2 levels.

**Table 3:** Initial laboratory values.

Characteristic	sST2 ≤ 53.42 ng/ml (n = 53)	sST2 > 53.42 ng/ml (n = 53)	p-value
Initial pH overall—median (IQR)	7.257 (7.163–7.329)	7.151 (7.023–7.276)	0.003
Initial lactate overall (mmol/l)—median (IQR)	3.58 (2.03–5.85)	5.74 (3.12–9.26)	0.003
Hemoglobin (g/dl)—median (IQR)	13.9 (12.9–14.6)	13.8 (12.5–15.0)	0.827
Leucocyte count (G/l)—median (IQR)	12.6 (10.3–15.4)	15.6 (12.9–20.2)	0.002
Platelet count (G/l)—median (IQR)	236 (203—277)	230 (189—311)	0.778
CRP (mg/dl)—median (IQR)	0.3 (0.2–0.8)	0.5 (0.2–1.5)	0.392
Serum creatinine (mg/dl)—median (IQR)	1.19 (1.02–1.37)	1.28 (1.15–1.40)	0.099
LDL (mg/dl)—median (IQR)	83 (54–111)	65 (44–97)	0.132
Hba1c (%)—median (IQR)	5.5 (5.3–5.7)	5.5 (5.3–5.9)	0.645

*CRP* C-reactive protein, *IQR* interquartile range, *LDL* low-density lipoprotein.

**Figure 2:**
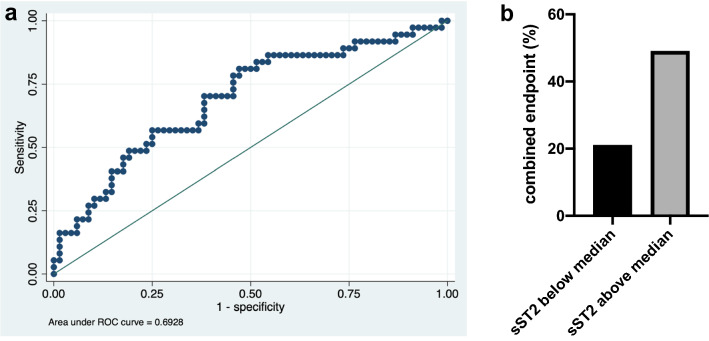
(**a**) ROC (receiver operating characteristic) curve for diagnostic performance of sST2 24 h after resuscitation for the primary end point (death or CPC > 2 at 6 months; optimal cut-off value 46.15 ng/ml; sensitivity 81%, specificity 53%, AUC 0.69). (**b**) Bar graph of combined end-point (death or CPC > 2 after 6 months) in patients with sST2-levels below and above the median (53.42 ng/ml) 24 h after ICU admission (21% vs. 49%, OR 3.59, 95% CI 1.52–8.45, p = 0.003).

**Figure 3:**
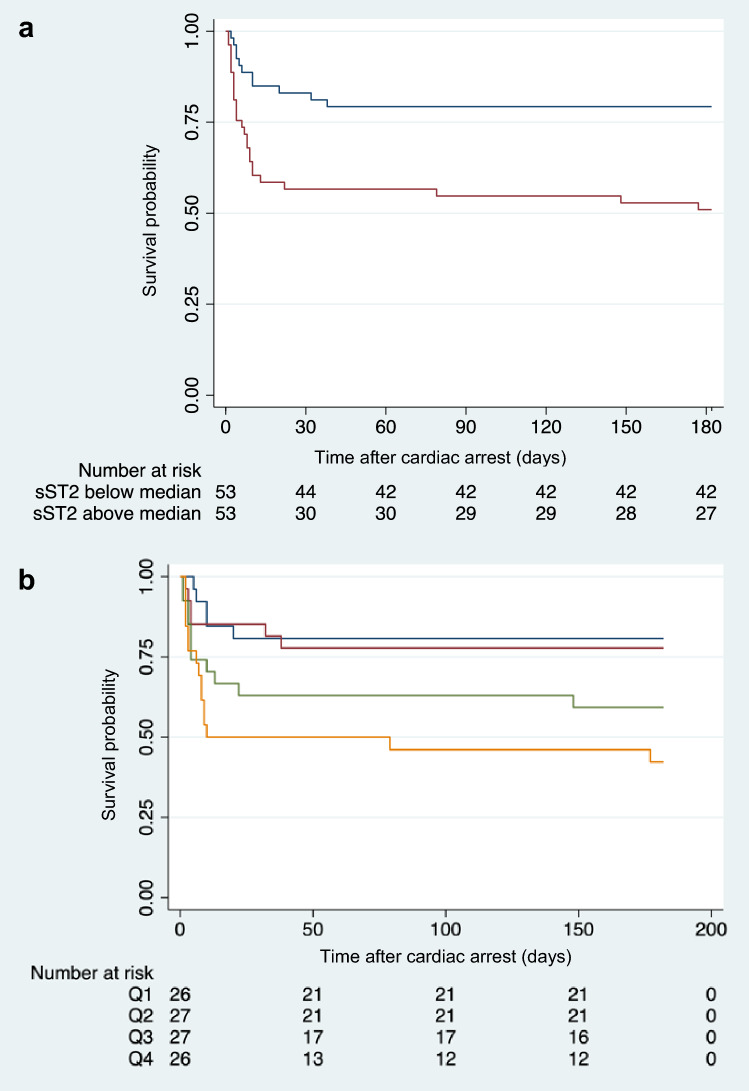
(**a**) Kaplan–Meier plot for patients with sST2-levels below (red line) and above the median (blue line; median sST2-level 24 h after admission: 53.42 ng/ml). (**b**) Kaplan–Meier plot for patients stratified by sST2-level quartiles (blue line: quartile 1, sST2 < 34.68 ng/ml; red line: quartile 2, sST2 ≥ 34.68 ng/ml and < 53.42 ng/ml; green line: quartile 3, sST2 ≥ 53.42 ng/ml and < 93.57 ng/ml; yellow line: quartile 4, sST2 ≥ 93.57 ng/ml).

Elevated sST2-levels 24 h after ICU admission were associated with a higher risk of the combined endpoint (death or CPC > 2) at 6 months (OR 1.011, 95% CI 1.004–1.019, p = 0.004). The optimal cut-off value for serum sST2 was 46.15 ng/ml (sensitivity 81%, specificity 53%, AUC 0.69). The corresponding ROC (receiver operating characteristic) curve is shown in Fig. 2a. There were no survivors with poor neurological outcome (CPC > 2) at 6 months. The association was significant for the secondary endpoint of death after 30 days (OR 1.011, 95% CI 1.004–1.02, p = 0.004). SST2-levels > 53.42 ng/ml were associated with higher odds for the primary endpoint (21% vs. 49%, OR 3.59, 95% CI 1.52–8.45, p = 0.003; see Fig. 2b). SST2 levels above the median were also associated with higher risk of death at 30 days (OR 3.75, 95% CI 1.52–9.21; see Fig. 3a). By dividing the cohort into four groups (stratified by quartiles), a graded association of sST2 and the primary endpoint was shown (see Fig. 3b). In the multivariable model, the association of both sST2 as a continuous variable (aOR 1.09 95% CI 1.001–1.018, p = 0.02) as well as sST2 above the median (aOR 2.95 95% CI 1.11–7.83, p = 0.03) with the primary outcome remained after adjustment for potential confounders. Neither IL-33 levels (p = 0.35) nor IL-33/sST2 ratio (p = 0.64) 24 h after admission were associated with the primary outcome.

## Discussion

In our observational prospective study of 106 consecutive patients after cardiopulmonary resuscitation, serum sST2-levels 24 h after hospital admission were associated with an increased risk of death at 30 days and 6 months.

In general, CA results not only in circulatory arrest, but also in microcirculatory shock with far-reaching metabolic, immunologic and coagulatory consequences^14^. After return of spontaneous circulation a generalized inflammatory reaction occurs, better known as Post-cardiac arrest syndrome (PCAS)^15^. PCAS is primarily caused by four main players, namely the cause of arrest and possible sustained sequela, full-body ischemia and reperfusion, as well as neurological and myocardial dysfunction^14^. Thus, metabolically active organs are particularly affected. In addition to cardiovascular and neural stress, coagulation pathways and the cortisol-stress axis are also involved^15^. Since both the origin of cardiac arrest and the exact onset of ischemia and reperfusion vary, it can be assumed that the subsequent defense response also varies in its strength and expression. A better understanding of the inflammatory pathways involved at the cellular level could facilitate clinical management and improve long-term outcome. Because circulatory arrest in CA affects all organ systems and the integrity of different vital organs is necessary for functionally intact survival, it is difficult to find a general biomarker for outcome prediction after CPR. After successful resuscitation, functional neurologic outcome is ultimately relevant, which is why resuscitation guidelines specifically mention biomarkers that may indicate neurological damage^2^. Nevertheless, the processes involved in PCAS are complex, and attention should be paid to prognostic tools aside from conventional markers.

ST2 was originally thought to be primarily relevant in inflammatory diseases, but its role as an indicator of myocardial stress and inflammation is now recognized. Its active transmembrane isoform, ST2L is believed to participate in the MyD88/NF-κB-pathway^3^. This pathway is essential regarding innate immunity activation via pathogen associated molecular pattern recognition^16^. Furthermore, it plays a role in regulation of T-helper cells type 2, regulatory T-cells, mastocytes and cells of the innate lymphoid system^17–19^. ST2L and IL-33 appear to have cardioprotective effects, whereas sST2 serves as a decoy-receptor for IL-33 (and therefore attenuates protective properties of ST2/IL-33) and was previously associated with worse outcomes^20,21^. Currently, sST2 is recognized as a relevant prognostic marker especially for patients with various cardiovascular diseases, such as ischemic heart disease and heart failure^22^. However, it is not a cardio-specific parameter because it is not only produced in cardiomyocytes but also in other organs such as lung and kidney^22,23^.

With regard to general patient characteristics, no significant differences were observed in our patient cohort with the exception of previously known CAD. Outcome analyses for sST2 in CAD have been performed in the past and higher baseline concentrations were associated with a risk of major adverse cardiac events in a recent meta-analysis of 17,432 individuals^24^. Nevertheless, this finding must be interpreted with caution, since some patients in our cohort were certainly overestimated based on their medical records (CAD present, but clinically insignificant) whereas other patients were underdiagnosed, because not all patients received coronary angiography after CPR. Another interesting finding of our study was that no correlation was found with ejection fraction obtained by echocardiography. Although all patients were examined by experienced physicians with sufficient training in echocardiography, not all measurements were performed in a standardized manner, as for example Simpson’s method. The possibility of reversible myocardial dysfunction must also be considered^25^. An interesting approach would be echocardiographic monitoring 72 h after CPR, as myocardial dysfunction is often transient and reversible within 48–72 h^26^. However, several other authors also did not report a correlation between sST2 levels and ejection fraction, as ejection fraction in contrast to diastolic function is known to be a weak outcome predictor after CPR^11,27–30^. Unfortunately, diastolic function was not recorded in our database.

Regarding CPR-specific findings, significantly more patients in the high-sST2 group received bystander CPR in our study. We do not have information on the quality (laypersons vs. professionals), downtime and duration of resuscitative measures, but this finding indicates the prognostic value of early CPR and witnessed arrest. In the study by Ristagno et al., no correlation with bystander CPR was shown for sST2 levels 0 h and 48 h after admission, although patients with shockable rhythms had lower sST2-levels than patients with non-shockable rhythms^12^. We could not fully reproduce this finding, although in our cohort, patients with higher sST2 levels (24 h after admission) were more likely to have pulseless electrical activity (PEA) as their initial rhythm. In a larger number of patients, this finding might have become statistically significant. In our study, patients with sST2 values above the median were also more likely to receive invasive measures like mechanical ventilation, antibiotics and hemodiafiltration and a higher rate of blood transfusions was observed, although this did not reach statistical significance. Regarding targeted temperature management (TTM), patients with higher sST2-levels received TTM more frequently. This is consistent with the results of Ristagno et al. 48 h after ROSC^12^. Bro-Jeppesen et al. found that concentrations of inflammatory cytokines (inter alia IL-1β) were associated with PCAS but no alteration of the inflammatory response by TTM was shown^31^. IL-1β-levels, which like ST2 belong to the IL-1 axis have been shown to correlate positively with sST2 in patients with acute decompensated heart failure^20^. However, no specific analysis on sST2-levels was carried out in the Bro-Jeppesen study^31^. Thus, clinically sicker patients (as reflected by the significantly higher SOFA-scores) most likely received TTM to a greater extent in our study. The decision to initiate TTM was made by the attending physician.

Regarding the laboratory values obtained, patients in the high sST2 group had both lower arterial pH- and higher lactate values after hospital admission. This is an expected finding, since both values have been associated with survival after CPR. Also, higher initial leucocyte counts (WBC) were found in the high sST2-group. Different observations have been made for WBC-count and sST2 in the past, as both positive and negative correlations with outcome have been observed in different diseases^32–34^. A more detailed evaluation including manual differential blood counts might be more informative, as the ST2-/IL-33 axis has a variety of interactions with different leukocyte subpopulations^17^. Interestingly, we did not find any relevant correlation with C-reactive protein (CRP) values, which we observed in a previous study in patients after transcatheter aortic valve replacement (TAVR)^35^. One possible explanation could be the timing of blood sampling, as Ristagno et al. demonstrated an association between higher sST2-, PTX3- and CRP-values 48 h after admission and poor outcome^12^. For future studies, serial blood sampling after admission would be useful as the release kinetics of sST2 after CA is currently unknown.

No correlation was found for IL-33 and our study endpoints either. Different release kinetics could be a possible explanation for this finding^36^. It is also consistent with the results of Zhuang et al., who observed increased IL-17- and IL-23- but not IL-33-levels in patients with poor outcome after PCAS^37^. In our own experience, IL-33 is not a strong direct predictor of outcome in cardiovascular disease. Nevertheless, a study with more participants and perhaps other sampling times would be necessary to reliably rule out an association between IL-33-levels and prognosis after CPR.

## Limitations

One limitation is the single-center character of this study, so it was not possible to include a larger number of patients. Information on downtime, quality of CPR, time to ROSC and duration of resuscitative measures is also lacking. Furthermore, conventional neuroprognostication markers such as NSE and S-100B protein, or novel markers like glial fibrillary acidic protein, serum tau protein and neurofilament light were not obtained by default in every patient. Because there was a relatively large group with a good neurological outcome in this patient population, the informative value of sST2 as a neuroprognostication marker is relatively low.

## Conclusion

In our prospective study of 106 patients after cardiopulmonary resuscitation, we found a positive correlation between serum sST2-levels and mortality at 30 days and 6 months after cardiac arrest. This study should contribute to current research on the inflammatory response after cardiac arrest and could serve as a basis for future studies.

## Supplementary Information

Supplementary Tables.

## Data Availability

All data relevant for this study will be given by the authors upon specific request without restriction.
