# Supplementary material for: Soluble suppression of tumorigenicity 2 as outcome predictor after cardiopulmonary resuscitation: an observational prospective study
Source: Sci Rep. 2021 Nov 5;11:21756. doi: 10.1038/s41598-021-01389-x (PMC8571342; doi:10.1038/s41598-021-01389-x)
Supplement: Supplementary file 1 — Supplementary Tables. [file 41598_2021_1389_MOESM1_ESM.docx]

**Supplement 1**

| **Characteristic** | **Q1 (n=26)** | **Q2 (n=27)** | **Q3 (n=27)** | **Q4 (n=26)** | **p-Value** |
| --- | --- | --- | --- | --- | --- |
| Age (yrs.) – median (IQR) | 64 (54 - 74) | 66 (58 - 74) | 66 (54 - 76) | 63 (53 - 70) | 0.63 |
| Male sex – no. (%) | 18 (69) | 20 (74) | 19 (70) | 19 (73) | 0.98 |
| BMI (kg/m^2^) – median (IQR) | 25 (25 - 28) | 26 (24 - 29) | 28 (26 - 29) | 25 (24 - 28) | 0.15 |
| Arterial hypertension – no. (%) | 17 (74) | 20 (91) | 23 (96) | 11 (58) | 0.007 |
| Hyperlipidemia – no. (%) | 16 (64) | 17 (63) | 15 (56) | 9 (36) | 0.16 |
| Diabetes mellitus – no. (%) | 5 (20) | 2 (8) | 6 (24) | 6 (25) | 0.37 |
| COPD – no. (%) | 4 (15) | 3 (11) | 3 (11) | 3 (12) | 0.96 |
| OSA – no. (%) | 4 (15) | 1 (4) | 1 (4) | 1 (4) | 0.23 |
| CKD – no. (%) | 9 (35) | 2 (7) | 6 (22) | 3 (12) | 0.059 |
| History of CAD – no. (%) | 8 (31) | 5 (19) | 2 (7) | 3 (12) | 0.12 |
| History of cardiac surgery – no. (%) | 3 (12) | 2 (8) | 1 (4) | 2 (8) | 0.90 |
| Ejection fraction after admission – no. (%) | - | - | - | - | 0.36 |
| Normal | 7 (27) | 2 (7) | 3 (11) | 5 (19) | - |
| Mildly abnormal | 4 (15) | 5 (19) | 10 (37) | 6 (23) | - |
| Moderately abnormal | 9 (35) | 11 (41) | 9 (33) | 6 (23) | - |
| Severely abnormal | 6 (23) | 9 (33) | 5 (19) | 9 (35) | - |

**Supplementary Table 1:** General patient characteristics. Abbreviations: BMI: body mass index; CAD: coronary artery disease; CKD: chronic kidney disease; COPD: chronic obstructive pulmonary disease; IQR: interquartile range; no.: number; OSA: obstructive sleep apnea; yrs.: years; ranges of percentage for left ventricular ejection fraction: "normal": males 52-72%, females 54-74%; "mildly abnormal": males 41-51%, females 41-53%; "moderately abnormal": both sexes 30-40%; "severely abnormal": both sexes <30%. Q1/quartile 1: sST2<34.68 ng/ml; Q2/quartile 2: sST2≥34.68 ng/ml & <53.42 ng/ml; Q3/quartile 3: sST2≥53.42 ng/ml & <93.57 ng/ml; Q4/quartile 4 sST2≥93.57 ng/ml;

| **Characteristic** | **Q1 (n=26)** | **Q2 (n=27)** | **Q3 (n=27)** | **Q4 (n=26)** | **p-Value** |
| --- | --- | --- | --- | --- | --- |
| OHCA – no. (%) | 22 (85) | 23 (85) | 23 (85) | 22 (85) | 1.00 |
| Bystander CPR – no. (%) | - | - | - | - | 0.38 |
| Yes | 11 (61) | 15 (71) | 7 (41) | 8 (36) | - |
| No | 2 (11) | 2 (10) | 4 (24) | 6 (27) | - |
| In-hospital | 3 (17) | 4 (19) | 3 (18) | 4 (18) | - |
| During transport | 2 (11) | 0 (0) | 3 (18) | 4 (18) | - |
| Initial rhythm | - | - | - | - | 0.30 |
| Ventricular fibrillation | 20 (77) | 19 (79) | 19 (70) | 18 (69) | - |
| Asystole | 4 (15) | 3 (12) | 2 (7) | 6 (23) | - |
| PEA | 2 (8) | 1 (4) | 6 (22) | 2 (8) | - |
| Unknown/other | 0 (0) | 1 (4) | 0 (0) | 0 (0) | - |
| Admission directly to ICU – no. (%) | - | - | - | - | 0.17 |
| Yes | 2 (8) | 10 (37) | 5 (19) | 8 (31) | - |
| Via ER | 19 (73) | 15 (56) | 16 (59) | 15 (58) | - |
| Via Cath-lab | 5 (19) | 2 (7) | 6 (22) | 3 (12) | - |
| Systemic lysis – no. (%) | 2 (8) | 1 (4) | 3 (11) | 3 (12) | 0.71 |
| Coronary angiography – no. (%) | 21 (81) | 22 (81) | 19 (70) | 19 (73) | 0.71 |
| PCI – no. (%) | 12 (46) | 17 (63) | 15 (56) | 15 (58) | 0.66 |
| Mechanical ventilation – no. (%) | 20 (77) | 27 (100) | 27 (100) | 26 (100) | <0.001 |
| TTM – no. (%) | 10 (38) | 19 (70) | 24 (89) | 20 (77) | 0.001 |
| CVVHDF – no. (%) | 0 (0) | 0 (0) | 5 (19) | 5 (19) | 0.011 |
| Antibiotics – no. (%) | 18 (69) | 27 (100) | 27 (100) | 26 (100) | <0.001 |
| Blood transfusions – no. (%) | 3 (12) | 2 (7) | 4 (15) | 4 (15) | 0.80 |
| SOFA-Score – median (IQR) | 9 (7 – 10) | 11 (9 – 12) | 11 (10 – 12) | 12 (11 – 13) | <0.001 |

**Supplementary Table 2:** Patient characteristics regarding CPR and post-resuscitation care. Abbreviations: CPR: cardiopulmonary resuscitation; CVVHDF: continuous veno-venous hemodiafiltration; ER: emergency room; ICU: intensive care unit; IQR: interquartile range; OHCA: out of hospital cardiac arrest; PCI: percutaneous coronary intervention; PEA: pulseless electrical activity; SOFA: severity of organ failure assessment; TTM: targeted temperature management; Q1/quartile 1: sST2<34.68 ng/ml; Q2/quartile 2: sST2≥34.68 ng/ml & <53.42 ng/ml; Q3/quartile 3: sST2≥53.42 ng/ml & <93.57 ng/ml; Q4/quartile 4 sST2≥93.57 ng/ml;

| **Characteristic** | **Q1 (n=26)** | **Q2 (n=27)** | **Q3 (n=27)** | **Q4 (n=26)** | **p-Value** |
| --- | --- | --- | --- | --- | --- |
| Initial pH overall – median (IQR) | 7.256 (7.130 – 7.307) | 7.257 (7.185 – 7.344) | 7.193 (7.060 – 7.295) | 7.130 (6.884 – 7.242) | 0.008 |
| Initial lactate overall (mmol/l) – median (IQR) | 3.68 (2.22 – 5.81) | 3.00 (1.93 – 6.46) | 5.36 (2.98 – 7.99) | 6.14 (3.31 – 10.05) | 0.022 |
| Hemoglobin (g/dl) – median (IQR) | 13.3. (12.5 – 14.2) | 14.3 (13.3 – 14.9) | 14.5 (13.0 – 15.2) | 13.3 (12.3 – 14.6) | 0.13 |
| Leucocyte count (G/l) – median (IQR) | 12.3 (10.5 – 16.2) | 13.3. (11.1 – 15.4) | 15.0 (12.6 – 19.8) | 16.9 (13.4 – 21.5) | 0.014 |
| Platelet count (G/l) – median (IQR) | 228 (188 – 295) | 240 (208 – 274) | 259 (210 – 329) | 224 (184 – 266) | 0.35 |
| CRP (mg/dl) – median (IQR) | 0.4 (0.1 – 0.6) | 0.3 (0.2 – 1.8) | 0.4 (0.2 – 1.0) | 0.6 (0.1 – 1.7) | 0.69 |
| Serum creatinine (mg/dl) – median (IQR) | 1.2 (1.0 – 1.4) | 1.2 (1.0 – 1.3) | 1.2 (1.2 – 1.4) | 1.3 (1.1 – 1.5) | 0.35 |
| LDL (mg/dl) – median (IQR) | 85 (53 – 118) | 78 (59 – 106) | 65 (50 – 114) | 64 (42 – 93) | 0.36 |
| Hba1c (%)– median (IQR) | 5.5 (5.3 – 5.8) | 5.5 (5.4 – 5.7) | 5.5 (5.4 – 5.9) | 5.6 (5.2 – 5.9) | 0.97 |

**Supplementary Table 3:** Initial laboratory values. Abbreviations: CRP: C-reactive protein; IQR: interquartile range; LDL: low-density lipoprotein; Q1/quartile 1: sST2<34.68 ng/ml; Q2/quartile 2: sST2≥34.68 ng/ml & <53.42 ng/ml; Q3/quartile 3: sST2≥53.42 ng/ml & <93.57 ng/ml; Q4/quartile 4 sST2≥93.57 ng/ml;
